# Impact of Nitrogen on the Selective Closure of Stacking Faults in 3C-SiC

**DOI:** 10.1021/acs.cgd.2c00515

**Published:** 2022-06-29

**Authors:** Cristiano Calabretta, Viviana Scuderi, Corrado Bongiorno, Annalisa Cannizzaro, Ruggero Anzalone, Lucia Calcagno, Marco Mauceri, Danilo Crippa, Simona Boninelli, Francesco La Via

**Affiliations:** † CNR-IMM, VIII Strada, 5, 95121 Catania, Italy; ‡ STMicroelectronics, Stradale Primosole, 50, 95121 Catania, Italy; § Dipartimento di Fisica e Astronomia, Università di Catania, Via S. Sofia 64, I-95123 Catania, Italy; ∥ LPE, XVI Strada, 95121 Catania, Italy; ⊥ LPE, via Falzarego 8, 20021 Baranzate (MI), Italy

## Abstract

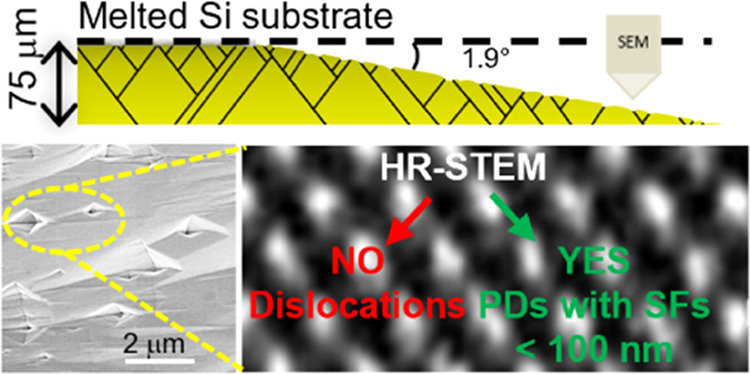

Despite the promising properties, the problem of cubic silicon carbide (3C-SiC) heteroepitaxy on silicon has not yet been resolved and its use in microelectronics is limited by the presence of extensive defects. In this paper, we used microphotoluminescence (μ-PL), molten KOH etching, and high-resolution scanning transmission electron microscopy (HRSTEM) to investigate the effect of nitrogen doping on the distribution of stacking faults (SFs) and assess how increasing dosages of nitrogen during chemical vapor deposition (CVD) growth inhibits the development of SFs. An innovative angle-resolved SEM observation approach of molten KOH-etched samples resulted in detailed statistics on the density of the different types of defects as a function of the growth thickness of 3C-SiC free-standing samples with varied levels of nitrogen doping. Moreover, we proceeded to shed light on defects revealed by a diamond-shaped pit. In the past, they were conventionally associated with dislocations (Ds) due to what happens in 4H-SiC, where the formation of pits is always linked with the presence of Ds. In this work, the supposed Ds were observed at high magnification (by HRSTEM), demonstrating that principally they are partial dislocations (PDs) that delimit an SF, whose development and propagation are suppressed by the presence of nitrogen. These results were compared with VESTA simulations, which allowed to simulate the 3C-SiC lattice to design two 3C-lattice domains delimited by different types of SFs. In addition, through previous experimental evidence, a preferential impact of nitrogen on the closure of 6H-like SFs was observed as compared to 4H-like SFs.

## Abstract 2

Innovative angle-resolved SEM analysis of molten KOH etching provides a portrait of the evolution of defects as a function of 3C-SiC growth thickness. Nitrogen doping is remarkably effective in limiting the density of SFs. Diamond-shaped etch pits were also followed and prevalently identified through HAADF-STEM analysis as partial dislocations of SFs with an extension of less than 100 nm.

## Introduction

Emerging wide band gap (WBG) semiconductor devices based on silicon carbide (SiC) and gallium nitride (GaN) can revolutionize power electronics due to their extraordinary properties^1^ compared to standard silicon-based devices. In particular, 3C-SiC can find application not only in power electronics^2,3^ but also in new emerging fields, such as photocatalysis,^4^ water splitting,^5^ and biological applications.^6^

However, despite the promising properties, the problem of 3C-SiC heteroepitaxy on silicon has not yet been resolved and its use in microelectronics is limited by the presence of extensive defects. Defects such as stacking faults (SFs), partial dislocations (PDs), and inverted domain boundaries (IDBs) negatively affect the electrical and mechanical properties of the material. IDBs are often called antiphase boundaries (APB), and many works are present in the literature regarding their formation,^7,8^ development,^9^ and the detrimental effects that they have on the electrical properties of the material.^10,11^ Stacking faults are planar defects produced by either an excess or a lack of a single Si–C bilayer during the growth, and they consist of a wrong stacking order with respect to the 3C-SiC one.^12^ They are generated at the interface due to the mismatch between the crystal lattice of silicon and silicon carbide, and they propagate into the SiC epilayer to the surface, influencing the mechanical and electrical properties of the material.

Two PDs delimit each SF, and they limit the wrong sequence plan from the perfect 3C sequence regions. A dislocation (D) is a linear crystallographic defect within a crystal structure. It contains an abrupt change in the arrangement of atoms. Ds will decompose into PDs if the energy state of the sum of the partials is less than the energy state of the original dislocation.^13^ Dislocation complexes delimiting multiple SFs were shown to generate intragap electronic states in the material, causing leakage currents.^11^ In addition, the expansion or shrinkage of the SF is driven by the energy and kinetics of the PDs. Hence, the shape, direction, and stability of the PDs are important.^14−16^

In previous work,^17^ we observed that nitrogen doping concentration has a deep impact on the density and the average length of the SFs that reach the surface. As the nitrogen concentration increases, the average length of the SFs increases, from a value of 2 μm (intrinsic sample) to 5 μm (5.8 × 10^19^ atom/cm^3^), and the density decreases, from 2050 cm^–1^ (intrinsic sample) to 244 cm^–1^ (5.8 × 10^19^ atom/cm^3^). This result suggests that near the interface with the removed silicon there is a different concentration of defects as a function of the dopant concentration. More investigations should be carried out, particularly in cross sections, to determine the role of doping concentration in the density, shape, and stability of PDs because the expansion or shrinkage of SFs is driven by the energetics and kinetics of the PDs. In addition, much of the information we have on dislocations in 3C-SiC is reconstructed based on what happens in 4H-SiC. Here, after chemical etching, hexagonal etch pits are formed at the locations of dislocations due to the deformation field.^18^

In this study, we used microphotoluminescence (μ-PL), molten KOH etching, and high-resolution scanning transmission electron microscopy (HRSTEM) to investigate the effect of nitrogen doping on the distribution of stacking faults (SFs) and assess how increasing dosages of nitrogen during chemical vapor deposition (CVD) growth inhibits the development of SFs. An innovative angle-resolved SEM observation approach of molten KOH-etched samples resulted in a detailed statistic on the density of the different types of defects as a function of the growth thickness of 3C-SiC free-standing samples with varying levels of nitrogen doping. Moreover, we proceeded to shed light on defects revealed by diamond-shaped pits. In the past, they were conventionally associated with Ds due to what occurs in 4H-SiC, where the formation of pits is always linked to the presence of Ds. In this work, the supposed Ds were observed at high magnification (by HRSTEM), demonstrating that principally they are PDs that delimit an SF, whose development and propagation are suppressed by the presence of nitrogen. These results were compared with VESTA simulations, which allowed to simulate the 3C-SiC lattice to design two 3C-lattice domains delimited by different types of SFs. In addition, through previous experimental evidence, a preferential impact of nitrogen on the closure of 6H-like SFs was observed as compared to 4H-like SFs.

## Materials and Methods

3C-SiC was grown heteroepitaxially using chemical vapor deposition (CVD) in a horizontal hot-wall reactor (ACIS M10) at the LPE industry (Catania, Italy) on a Si (100) 4° off-axis substrate. The CVD growth process was performed at a pressure and temperature of 100 mbar and 1370 °C, respectively. At this temperature, the growth of the 3C-SiC takes place, with three different growth rates of 3, 6, and 33 μm/h. The gases used during the growth were trichlorosilane (TCS) and ethylene (C_2_H_4_) as silicon and carbon precursors, respectively (the C/Si ratio was changed from 1.12 to 0.7). Hydrogen (H_2_) was used as a carrier gas and constant nitrogen flux, with a value of 0 (intrinsic), 300, 800, or 1600 sccm, was introduced within the chamber to dope the samples. After the growth of a 75 μm thick layer, the silicon substrate was melted inside the CVD chamber at a temperature of 1650 °C to achieve a 3C-SiC free-standing sample.^19^ For nitrogen, atomic concentration values were obtained from calibration curves acquired through secondary-ion mass spectrometry (SIMS) analysis, and the extracted results were 1.2 × 10^19^, 2.9 × 10^19^, and 5.8 × 10^19^ atom/cm^3^.

The carrier concentrations were also measured by a four-point probe and Hall technique at room temperature (RT), and the samples, patterned to the van der Paw geometry, were square-shaped (1 cm^2^). The determined carrier concentration values in the three previous samples were 0.13 × 10^19^, 0.75 × 10^19^, and 0.95 × 10^19^ atom/cm^3^, respectively. The mobility value was in the range of 100–60 cm^2^V/s. The values of carrier concentration were rather lower than the atomic concentration extracted by SIMS analysis; the difference was related to the incomplete ionization of N-donor atoms at RT according to the literature.^18^ This point will be discussed in detail in the Results and Discussion Section.

Microphotoluminescence maps were acquired at room temperature using a Horiba Jobin Yvon HR800 spectrometer (Horiba, Lille, France) integrated system in a backscattering configuration. The excitation wavelength was supplied by a 325 nm He–Cd continuous-wave laser that was focalized on the sample by a 40× objective, with a numerical aperture (NA) of 0.5. The scattered light was dispersed by a 300 grooves/mm kinematic grating.

Before etching in KOH, the samples were mechanically thinned at an angle of 1.9°. Thus, it was possible to follow the distribution of defects as a function of depth. Etching in potassium hydroxide (KOH) was adopted for the evaluation of SFs and possible Ds. KOH etching was performed at 500 °C for 3 min. The densities were calculated based on the observation of scanning electron microscopy (SEM) images, operating at an acceleration voltage of 5 kV (Field-Emission SEM ZeissSupraTM25, Carl Zeiss NTS GmbH, Oberkochen, Germany). The SFs and possible Ds (diamond-shaped etch pits) were identified and counted using ImageJ software.

Transmission and scanning transmission electron microscopy (TEM, STEM) analyses were performed on a JEOL ARM200F probe Cs-corrected TEM, equipped with a cold FEG working at 200 kV. We operated with three detectors, acquiring three images contemporaneously: at low, medium, and high scattering angles. We followed the defects from the low magnification to the high resolution. A low-angle detector allowed us to have bright-field (BF) images, while a high-angle annular dark-field (HAADF) detector (with an inner semiangle of 80 mrad) was used to obtain the dark-field STEM images. In this configuration, the signal in the image is mainly related to the atomic number *Z*, allowing direct identification of the carbon and silicon atomic columns at a distance of 1.1 Å each other. The images were exported using digital micrograph software and analyzed with ImageJ software. We used ImageJ tools to denoise, smooth, and modify the contrast of the images. Images in “plan view” were acquired with a JEOL JEM2010F in TEM mode. Diffraction contrast was used in standard and two-beam configurations for the study of the SFs and PDs.

The 3C-SiC lattice was simulated by means of the three-dimensional (3D) visualization program VESTA for structural models to draw two 3C-lattice domains bounded by different stacking fault types.

## Results and Discussion

**Figure 1:**
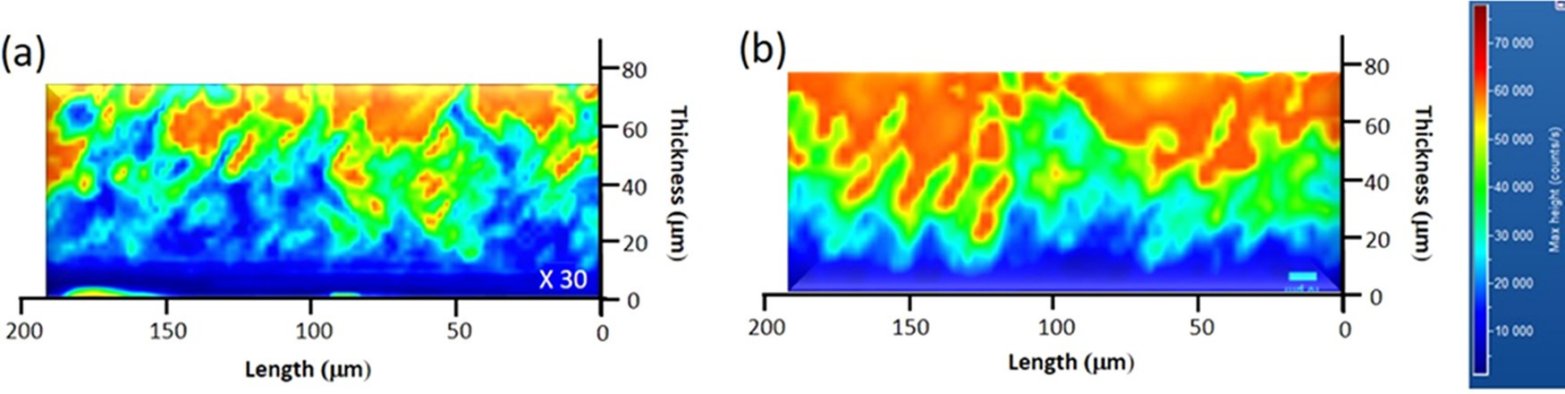
Room-temperature micro-PL map at 540 nm in cross section for (a) intrinsic and (b) 5.8 × 10^19^ atom/cm^3^ samples. For both images, the marker is 10 μm.

Figure 1 shows the room-temperature micro-PL map in correspondence to the 3C-SiC band-to-band emission signal at 540 nm acquired on a 3C-SiC cross section, for intrinsic (Figure 1a) and 5.8 × 10^19^ atom/cm^3^ (Figure 1b) samples. To rule out the impact of activated doping on the rise of radiative recombination processes, the intensity of the map acquired along the intrinsic sample was enhanced by a factor of 30 and normalized to the intensity of the doped sample’s emission. We can observe that the region next to the removed silicon interface (point 0 on the *Y*-axis) appears blue due to a lower intensity of the band-edge peak, about 10 000 counts/s. As we approach the surface, instead, the intensity of the map increases, revealing an emission of the band-edge peak of about 60 000 counts/s. For the intrinsic sample, the distribution of the intensity of the band-edge shows values on average lower compared to the 5.8 × 10^19^ atom/cm^3^ sample, as visible on the map. In particular, it is possible to find an emission characterized by 10 000 counts even near the surface, where the doped sample exhibits more than 60 000 counts.

SFs in 3C-SiC do not generate new PL peaks in the 450–900 nm region since they do not introduce levels inside the band gap.^20,21^ However, the variation of the band-edge peak intensity in the map provides a distribution of crystalline quality and thus of defects, along the sample section.

**Figure 2:**
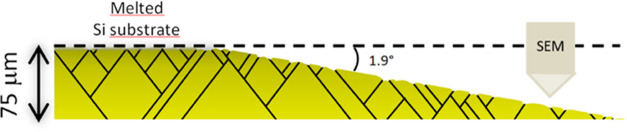
Scheme of a sample mechanically thinned at an angle of 1.9° and etched in KOH. The samples were etched from the backside.

To confirm this hypothesis, the samples were mechanically thinned at an angle of 1.9°. So, it was possible to follow the distribution of defects as a function of thickness after etching in KOH, through an in-plan analysis, in accordance with the scheme shown in Figure 2.

In fact, molten KOH etching can reveal such defects. Shibahara et al.^22^ etched a β-SiC sample grown on a Si(100) substrate in KOH at 600 °C. Etch pits due to Ds, SFs, and APBs were revealed as diamond-shaped pits, linear pits of various ratios of length and width, and random grooves, respectively.

**Figure 3:**
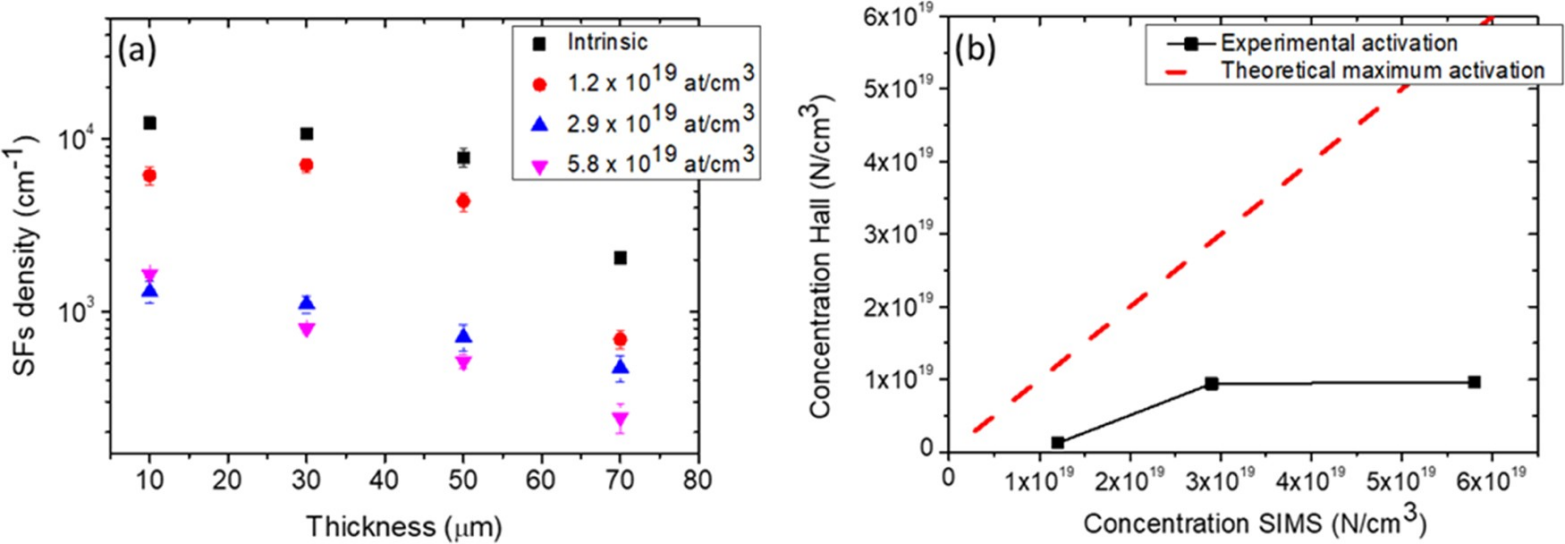
(a) Distribution of SFs vs thickness. Point 0 on the abscissa is the removed interface with the silicon. (b) Comparison between theoretical maximum activation and experimental activation.

The density of the SFs as a function of the thickness is plotted in Figure 3a.

Concerning the density of the SFs (Figure 3a), it was observed that by increasing the thickness the average density decreases for all samples. The counting of the defects was performed starting from a thickness of 10 μm away from the interface. This is due to the high concentration of defects in the region close to the removed silicon interface, which makes a clear discrimination between the different types of defects extremely challenging. Moving from 10 to 70 μm thickness, Figure 3a shows that the intrinsic sample (black squares) follows a constant concentration trend for the SFs whose experimental data revealed a density ranging from 1.2 × 10^4^ cm^–1^ at 10 μm to 9.7 × 10^3^ cm^–1^ at 50 μm. A significant reduction was observed in the last 20 μm. In fact, a density of 2.1 × 10^3^ cm^–1^ was measured on the surface of the sample (70 μm).

For sample 1.2 × 10^19^ atom/cm^3^ (red points), a density range from 6.2 × 10^3^ cm^–1^ at 10 μm to 6.9 × 10^2^ cm^–1^ at 70 μm was detected. For sample 2.9 × 10^19^ atom/cm^3^ (blue triangles), instead, the density was 1.3 × 10^3^ cm^–1^ at 10 μm and 4.7 × 10^2^ cm^–1^ at 70 μm. The increase in nitrogen concentration was responsible for a significant shift in the density curve of the SFs to lower values. In fact, as reported in the literature,^23,24^ nitrogen increased the formation energy of the small SFs, thus reducing their density in heavily doped samples.

At last, in the case of higher N_2_ flow (sample 5.8 × 10^19^ atom/cm^3^), the density of the SFs decreases from a value of 1.6 × 10^3^ cm^–1^ to 2.4 × 10^2^ cm^–1^ at 70 μm (magenta triangles). This trend (and absolute values) is comparable with that of sample 2.9 × 10^19^ atom/cm^3^.

To understand better the similarity of the values obtained for samples 2.9 × 10^19^ and 5.8 × 10^19^ atom/cm^3^, Figure 3b shows the comparison between the doping concentration measured by SIMS and the experimental activation of the samples obtained by Hall measurements. This comparison explains how not all of the nitrogen impurities introduced into the 3C-SiC film are substitutive with the carbon atoms. In fact, if all nitrogen impurities were substitutional, the experimental points of activation would lie on the bisector (dashed red line). Instead, the experimental points (black squares) lie outside the bisector, showing how a part of the nitrogen is taken up by the 3C-SiC film in an interstitial position. Interstitial nitrogen increases with increasing doping concentration during CVD growth. So, only substitutional nitrogen appears to play an active role in the closure of the SFs; in fact, the slopes and the final values of the blue and magenta points in Figure 3a are very close.

**Figure 4:**
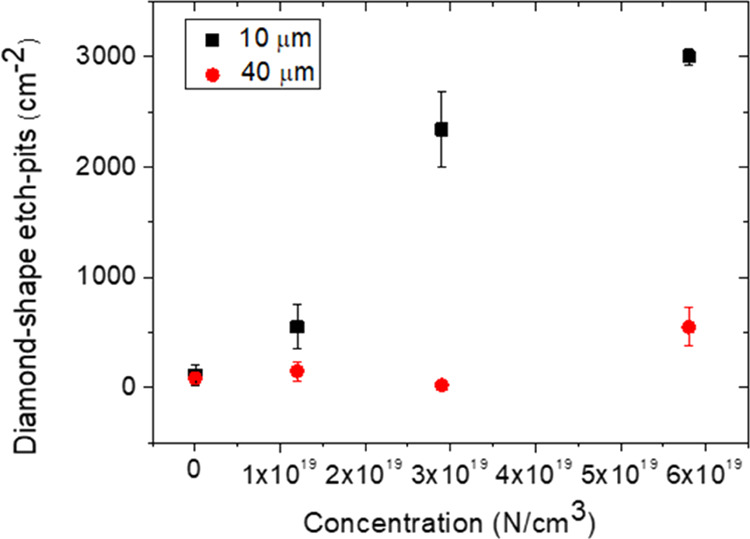
Diamond-shaped etched pits vs nitrogen concentration. For the intrinsic sample (2 × 10^16^ atom/cm^3^), the etched pit density is the same at 10 and 40 μm. From 40 to 70 μm, the concentration of etch pits is approximately constant for each nitrogen concentration.

Figure 4 shows the density of the diamond-shaped etched pits as a function of the nitrogen concentration. Generally, these pits were related to Ds.^22,25,26^

**Figure 5:**
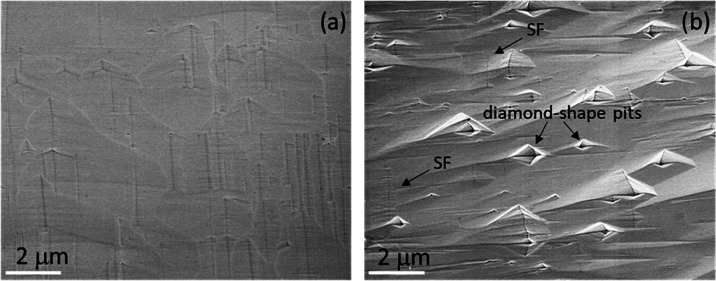
SEM images acquired at a thickness of 40 μm for (a) intrinsic sample and (b) 5.8 × 10^19^ atom/cm^3^ sample after etching in KOH.

For the intrinsic sample (2 × 10^16^ atom/cm^3^), we observe a constant value of about 120 cm^–2^, along all thicknesses. For all doped samples, instead, at about 10 μm from the removed silicon interface (black squares), the density of the etched pits is in the range between 5.5 × 10^2^ and 3.0 × 10^3^ cm^–2^, and it increases by enhancing the nitrogen concentration. At 10 μm, the trend of the experimental data is consistent with the SIMS analyses. In fact, as it can be seen in Figure 3b, by increasing the nitrogen concentration, the amount of interstitial nitrogen trapped by the film increases. Additionally, an increase in the defects identified by the diamond-shaped etched pits was observed. Therefore, their densities decrease with growing thickness, and they remain constant for thicknesses greater than 40 μm. Figure 5 shows as an example, the comparison between two images acquired at a thickness of 40 μm, for the intrinsic sample (Figure 5a) and the 5.8 × 10^19^ atom/cm^3^ sample (Figure 5b), following molten KOH etching.

In Figure 5a, we can observe the linear pits with various lengths and widths due to etched SFs. In Figure 5b, instead, in addition to the SFs, it is possible to observe the diamond-shaped pits generally related to Ds,^22,25,26^ as indicated by the arrows.

**Figure 6:**
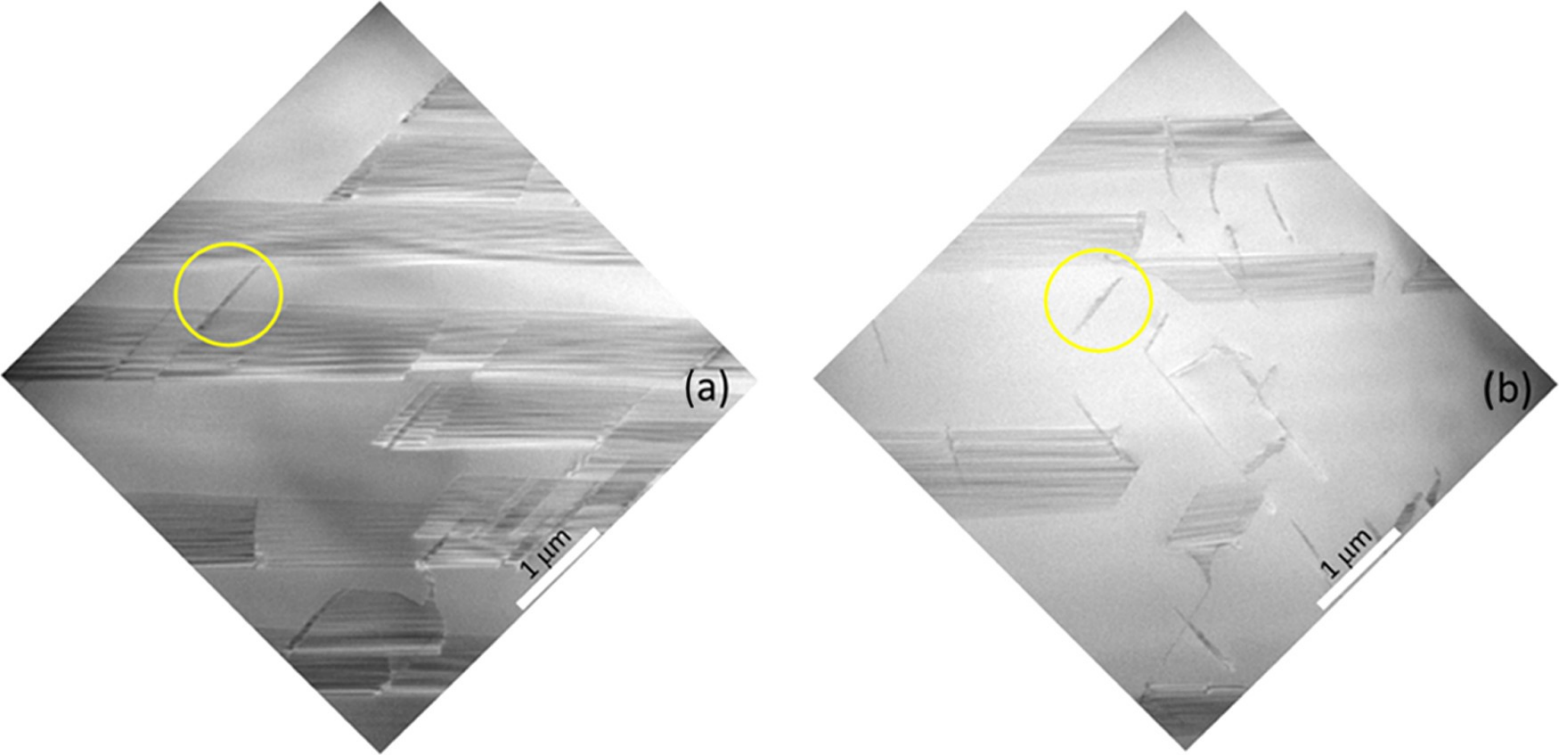
Cross-sectional TEM images acquired at a depth of 40 μm for (a) intrinsic sample and (b) 5.8 × 10^19^ atom/cm^3^ sample.

To understand better the significant difference in the density of the diamond-shaped pits (and related defects) between the intrinsic sample and the N-doped samples, TEM analyses were carried out in the cross section. Figure 6 shows a comparison between the intrinsic (Figure 6a) and 5.8 × 10^19^ atom/cm^3^ sample (Figure 6b). In particular, TEM images were acquired at a depth of 40 μm. In 3C-SiC, SFs always lie in the {111} planes.^20,27^ Two of the four {111} planes, the (1–11) and the (−111) ones, are perpendicular to the (110) observation plane. So, SFs appear as straight lines. Instead, the SFs lying in planes (111) and (−1–11) intersect the faces of the TEM lamellae and they appear in a trapezoidal shape. In our case, since the samples were grown off-axis, the SFs generated along the growth steps are mainly observed. These SFs lie on the (111) and (−1–11) planes, and they appear in a trapezoidal shape. For both samples, it is possible to observe SFs, with higher density in the intrinsic one. At the same time, a higher concentration of narrow defects (enclosed by yellow circles) can be observed in the 5.8 × 10^19^ atom/cm^3^ sample (Figure 6b).

Based on the results of experimental observations, Zimbone et al.^9^ observed that the PDs, which limit the wrong sequence plane of the SFs from the perfect 3C sequence regions, were characterized by peculiar families of line directions. By carefully observing the shape of the narrow defects in Figure 6a,b, they exhibit preferential directions. In particular, the directions are predominantly parallel to the PDs that delimit the SFs.

**Figure 7:**
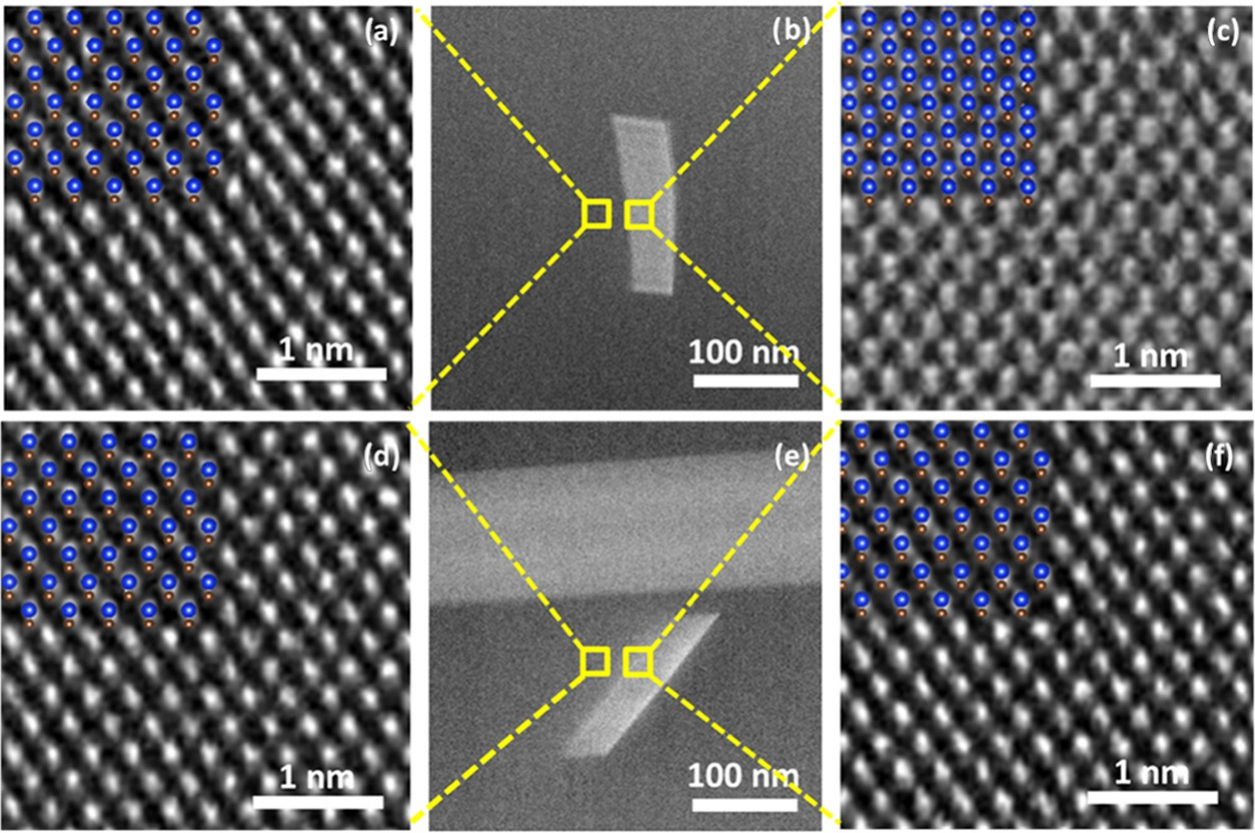
STEM images acquired on (b, e) different defects, (a, d) HRSTEM crystals, and (c, f) HRSTEM defects. Inset figures (a, c, d, f): a reconstruction of the atoms was shown to guide the reader’s eye. Atomic reconstruction was achieved with VESTA, which allowed us to simulate the 3C-SiC lattice to design two 3C-lattice domains delimited by different types of SFs.

In Figure 7b,e, two cross-sectional STEM images show at low resolution two narrow defects with preferential directions. The direction of observation of the crystal is the [110] zone axis.

In Figure 7a,c, high-resolution images acquired on the perfect 3C sequence regions and on a narrow defect, respectively, evidence that silicon and carbon in both regions are arranged differently. Silicon atoms have higher electron scattering cross sections than carbon atoms, so they are detectable as larger white halos, while smaller gray shoulders near Si indicate the C atoms. A reconstruction of the atoms is shown in Figure 7a,c to guide the reader’s eye. Atomic reconstruction was achieved with VESTA simulations, which allowed us to simulate the 3C-SiC lattice to design two 3C-lattice domains delimited by different types of SFs.

In the perfect crystal (Figure 7a), Si atoms (blue spheres) are above the C (red spheres) atoms. In the defect, instead, (Figure 7c), Si atoms are above another Si atom due to the distortion of the crystal introduced by an SF. In 3C-SiC, SFs can be intrinsic, extrinsic, and conservative depending on the number of atomic planes with the wrong orientation of the Si–C dimers. In particular, there are 2H-like, 4H-like, and 6H-like SFs, if they are made up of one, two, and three wrong atomic layers, respectively. By comparing the arrangement of the atoms with the Vesta model (both reported in Figure 7c), the spacing of the atoms can be attributed to the presence of a 4H-like SF taking in account the (110) observation plane.

Similarly, in Figure 7d,f, we report high-resolution images acquired on the perfect 3C sequence regions and on a further narrow defect, respectively, for another region of the sample. In this case, Si and C atoms have the same orientation in both images. This means that the defect shown in Figure 7e, for which a high magnification was acquired in Figure 7f, is a 6H-like SF.

We remind the reader that TEM images are acquired in transmission. This means that in the regions of the lamella without defects, the electron beam only passes through the perfect crystal before being collected. In the presence of an SF, the electron beam passes through a structure composed of the following elements before being collected: a crystal in front of the defect (1) and a crystal behind the defect (2). Crystal (2) can be shifted by one, two, or three atomic planes, with respect to crystal (1). This shift has a strong impact on high-resolution STEM images (e.g., Figure 7c), producing a new arrangement of the Si and C atoms within the crystalline matrix. Twinning nucleation can be measured by shearing two layers of (111) plane over a sequential 1/6 [112̅] direction. When HRSTEM is used to investigate the fault region, as shown in Figure 7c, the regular combination of Si–C doublets is disrupted by the visual alternation of Si–Si doublets. There is a 2*a*/3 [001] movement along the *c*-axis, where *a* is the lattice parameter, resulting in a shift between the crystal in front of the defect and the crystal behind the defect.

The only SF that allows realignment of crystal (2) with crystal (1) with respect to the *c*-axis is the 6H-like SF, which, by introducing the shift of three atomic planes (unitary shift), brings the whole system back to the tetrahedral stacking sequences of 3C (ABCABC).^12^ For further details, see the Supporting Information.

The results obtained through TEM analyses unequivocally show the limit of the macroscopic characterization of SiC samples. Concerning the density of the diamond-shaped etched pits, reported in Figure 4, the great difference between the intrinsic sample (black squares) and doped samples (120 cm^–2^ and in the range between 5.5 × 10^2^ and 3 × 10^3^ cm^–2^, respectively) must be reinterpreted. After chemical etching in KOH, a high concentration of diamond-shaped pits can be observed in the doped samples. For ages, these pits were associated with the presence of dislocations^22,25,26^ probably due to what happens in 4H-SiC, where the formation of pits is always linked to the presence of dislocations. Owing to the new experimental evidence, it is possible to state that many of these pits are associated with PDs, very close to each other, which delimits SFs with an extension (parallel to the base of the image, see Figure 7b, e) less than 100 nm. In 4H-SiC, the hexagonal etch pits are formed at the locations of the threading screw dislocations (TSDs) and the threading edge dislocations (TEDs), and the pit size is much larger for the TSD because the magnitude of the Burgers vector, and thereby the strain field, is much larger for TSDs.^18^ As a result, it is conceivable to conclude that the rate of the chemical etching is higher for PDs than that for SFs. Therefore, in the presence of PDs very close to each other (due to the nanometric extension of the SFs between the PDs), the etching proceeds faster in proximity to the PDs, forming rhomboid (diamond)-shaped pits.

At present, it is not possible to quantify the percentage of diamond-shaped pits associated with Ds and that associated with PDs by etching in KOH. However, as mentioned above, by carefully observing the shape of the narrow defects (Figure 6a,b), while some of them do not exhibit a preferential direction, most narrow defects share preferential directions, which are parallel to the PDs that delimit the SFs. It is reasonable to believe that all of the narrow defects that show a direction parallel to the PDs of the SFs are actually PDs that delimit SFs whose development was inhibited by nitrogen. Although the mechanism is not clear, the closure of the SFs can be attributed only to the presence of nitrogen since the amount of nitrogen is the only variable parameter among the samples. Theoretical calculations^23,24^ indicate that N doping around an SF increases the formation energy. However, the exact location of the nitrogen atoms is not clear. Models indicate that the effect of the dopant is rather short-ranged, about 10 Å. Furthermore, as reported by Lorenzzi et al.,^28^ since 3C has the smallest Si–C bond length along the [111] growth direction, important incorporation of nitrogen on the (111) oriented surface stabilizes the cubic polytype along this preferential direction. High nitrogen incorporation induces changes in internal Si–C stoichiometry. The substitutional incorporation of N atoms on C sites, from a macroscopic point of view, encourages an increase of the N_Si_/N_C_ ratio in the grown material (where N_Si_/N_C_ are the number of silicon atoms and carbon atoms). Any increase in the N_Si_/N_C_ ratio should be favorable to 3C-SiC.^29^

In previous work,^30^ we observed that in N-doped 3C-SiC samples, the SFs have an unexpected distribution along the thickness of the sample. In fact, even if 6H-like SF is considered the most common inclusion of other polytypes in 3C-SiC,^31^ due to its lower formation energy than 4H-like,^12^ we observed the presence of 6H-like SFs only in the first 15 μm of the film (from the removed Si interface). Instead, the presence of 4H-like SFs is detected in the first 20–25 μm of the film. On the basis of these new pieces of experimental evidence, it is possible to hypothesize that nitrogen has a selective role in the closure of SFs and, in particular, promotes the closure of 6H-like SFs.

**Figure 8:**
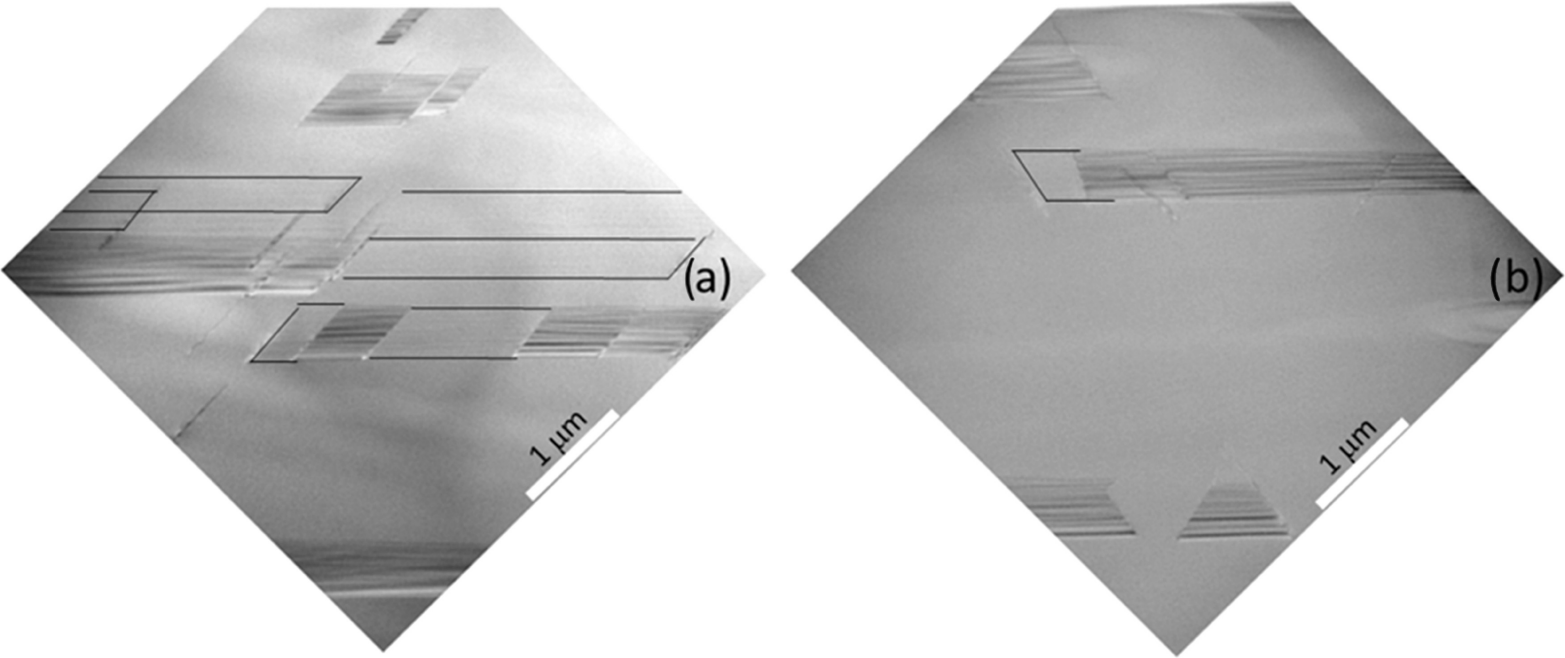
Cross-sectional TEM images acquired at the surface for (a) intrinsic sample and (b) 5.8 × 10^19^ atom/cm^3^ sample.

Figure 8 reports a comparison between the (a) intrinsic and (b) 5.8 × 10^19^ atom/cm^3^ sample by acquiring the TEM images (in cross section) near the surface. At first glance, it would seem that the two samples show a quite similar density of SFs.

However, on closer analysis, in Figure 8a, there are some SFs with very weak contrast. Due to the size of the image, the outlines of these SFs were marked with black lines to help the reader. As said previously, TEM images are acquired in transmission on an FCC crystal, where the fault vectors are shear vectors of type 1/6 <112> and as a result the phase angle in the electron wave amplitudes across the plane of the fault involves a series of three recurring values.^32^ The 6H-like SF, by introducing the displacement of three atomic planes and by bringing the whole system back to the 3C tetrahedral stacking sequences (ABCABC), determines a barely visible contrast in the image for this magnification. In fact, the electron beam along its path only encounters three nonaligned planes (those of the defect). Indeed, in this specific case, the total shear displacement is a proper translation vector of the lattice and thus the phase difference between the transmitted and the diffracted beam is an integer multiple of 2π and no contrast results.^32^ Introducing the displacement of two atomic planes, the 4H-like SF induces a shift between the two crystals (before and after the SF) that involves a nonunitary translation vector. This produces a high phase contrast in the image. In this case, the electron beam along its path encounters two crystals shifted relative to each other. The presence of SFs characterized by a weak contrast is abundant on the surface of the intrinsic sample (Figure 8a), while it is difficult to observe them by increasing the concentration of nitrogen in the sample (Figure 8b). As a result, it is noticeable that doping has a strong selective effect on the topology of SFs, making 6H-like SFs particularly unfavorable and preventing their spread in the crystal.

Theoretical investigation of polytypes in SiC^33^ suggests that the most stable structures are 6H and 4H in the presence of Si vacancies and C vacancies, respectively. Furthermore, substitutional nitrogen for C is energetically easier to form than substitutional nitrogen for Si and it stabilizes 3C compared to other polytypes.

## Conclusions

This paper provides a comprehensive investigation of the role of nitrogen in 3C-SiC epitaxial layers. The crystal is highly sensitive to N doping concentration. Concerning the density of the SFs, it was observed that by increasing the thickness the average density decreases for all samples. In particular, the intrinsic sample shows the concentration of the SFs ranging from 1.2 × 10^4^ cm^–1^ at 10 μm to 2.1 × 10^3^ cm^–1^ on the surface of the sample (70 μm), while in the case of higher N_2_ flow (sample 5.8 × 10^19^ atom/cm^3^), the density of the SFs ranges from a value of 1.6 × 10^3^ to 2.4 × 10^2^ cm^–1^ at 70 μm. Furthermore, the increase in nitrogen concentration is responsible for shifting the SF density curve to lower values. In fact, nitrogen increases the formation energy of the SFs, thus reducing their density in heavily doped samples.

About the density of the diamond-shaped etched pits, generally attributed to the presence of dislocations, for the intrinsic sample (2 × 10^16^ atom/cm^3^), we observe a constant value of about 120 cm^–2^ along all thicknesses. For all doped samples, instead, at about 10 μm from the removed silicon, the density of the etched pits is in the range between 5.5 × 10^2^ and 3.0 × 10^3^ cm^–2^, and it increases by increasing nitrogen concentration. At the same time, the density of diamond-shaped etched pits rapidly decreases during the growth. In contrast to the literature, which has always associated diamond-shaped etched pits with dislocations, STEM analysis allowed us to determine that many of the diamond-shaped etched pits are associated with PDs, very close to each other, which delimit SFs with an extension of less than 100 nm. The closure of the SFs can be attributed to the presence of nitrogen, and it was possible to assume that nitrogen has a selective role in the closure of SFs, favouring the closure of 6H-like SFs.

## Supporting Information Available

Atomic reconstruction achieved with VESTA, which allowed to simulate two 3C-lattice domains delimited by a 4H-like SF, and VESTA simulation of the three-layer SF displayed along the zone axis (0–10) (PDF)
