# Supplementary material for: Impact of Nitrogen on the Selective Closure of Stacking Faults in 3C-SiC
Source: Cryst Growth Des. 2022 Jun 29;22(8):4996–5003. doi: 10.1021/acs.cgd.2c00515 (PMC9354508; doi:10.1021/acs.cgd.2c00515)
Supplement: Supplementary file 1 — cg2c00515_si_001.pdf [file cg2c00515_si_001.pdf]

Supporting Information:

Impact of Nitrogen on the Selective Closure of Stacking Faults in 3C-SiC.

*Cristiano Calabretta [§], Viviana Scuderi [§, *], Corrado Bongiorno [§], Annalisa Cannizzaro [§], Ruggero Anzalone [ǂ], Lucia Calcagno [†], Marco Mauceri [‡], Danilo Crippa [□], Simona Boninelli [§], and Francesco La Via [§]*

AUTHOR ADDRESS

[§] CNR-IMM, VIII Strada, 5, 95121 Catania, Italy

[ǂ] STMicroelectronics, Stradale Primosole, 50, 95121 Catania, Italy

[†] Dipartimento di Fisica e Astronomia, Università di Catania, Via S. Sofia 64, I-95123 Catania, Italy

[‡] LPE, XVI Strada, 95121 Catania, Italy

[□] LPE via Falzarego 8, 20021 Baranzate (MI), Italy

*Corresponding author: viviana.scuderi@imm.cnr.it

S1 VESTA simulation

In order to facilitate the understanding of Figures 7(c) and 7(f) we propose the VESTA simulations of the HRSTEM acquisitions rotated by 90° around the axis [001], so as to schematically display both SFs of Figure 7 in section along the zone axis [1-10].

Following the rotation, the relative configuration of the Si-C dimer atoms is inverted, displaying Si upwards and C downwards. Figure S1 refers to the HRSTEM shown in Figure 7(c).

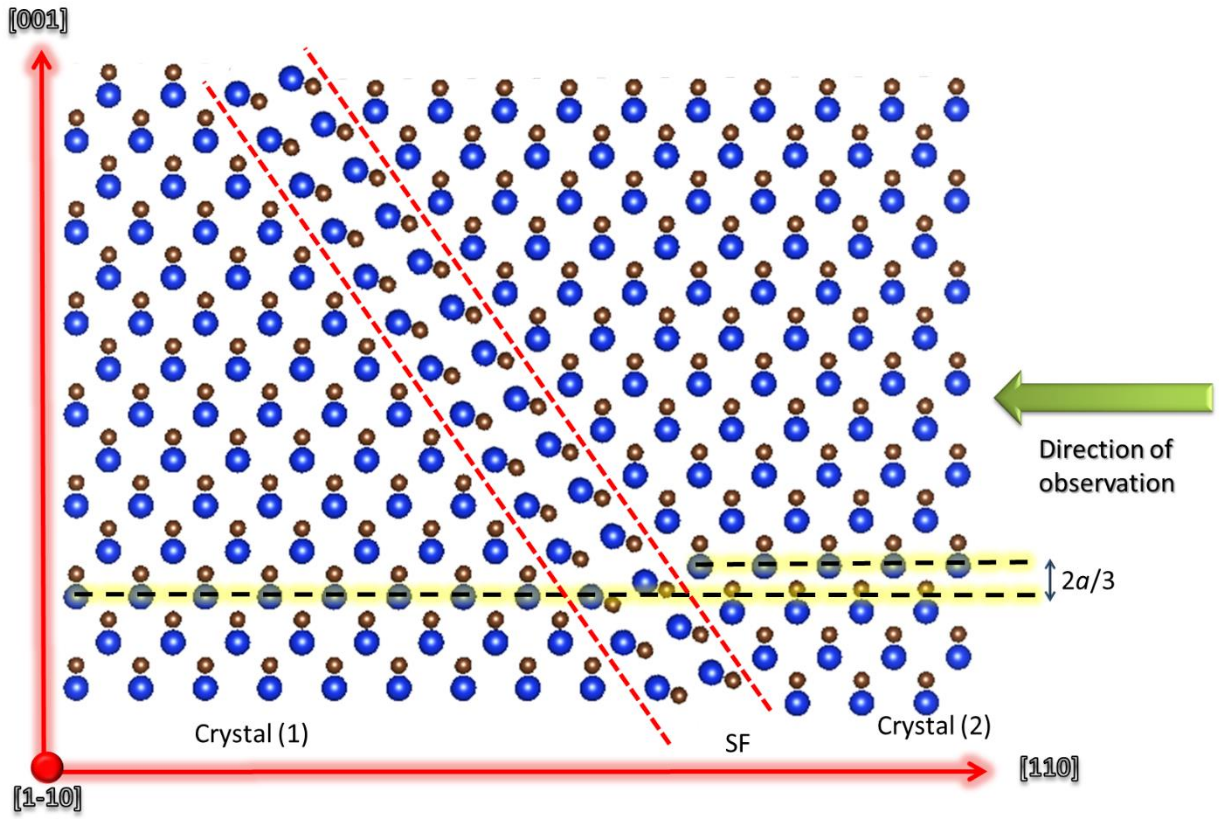

Figure S1. Atomic reconstruction was achieved with VESTA, which allowed to simulate two 3C-lattice domains delimited by a 4H-like SF. The Si atoms are represented by the blue spheres, while the C atoms are represented by the red spheres.

As can be seen from the black dashed lines under this configuration, the crystal (1) and the crystal (2) are displaced by "2*a*/3" along the [001] axis due to the presence of the 4H-like SF. If we switch the observation direction by adopting the [110] zone axis as indicated by the green arrow, the configuration of Figure 7(c) is finally achieved. Where Si atoms are above another Si atom due to the distortion of the crystal introduced by a SF. Indeed, the presence of the 2 layers related to SF is visualized optically by the formation of Si-Si doublets due to the overlap along the direction of observation of the two shifted crystals.

Figure S2 shows the VESTA simulation of the 3-layer SF (namely 6H-like SF) of Figure 7(d) displayed along the zone axis [1-10].

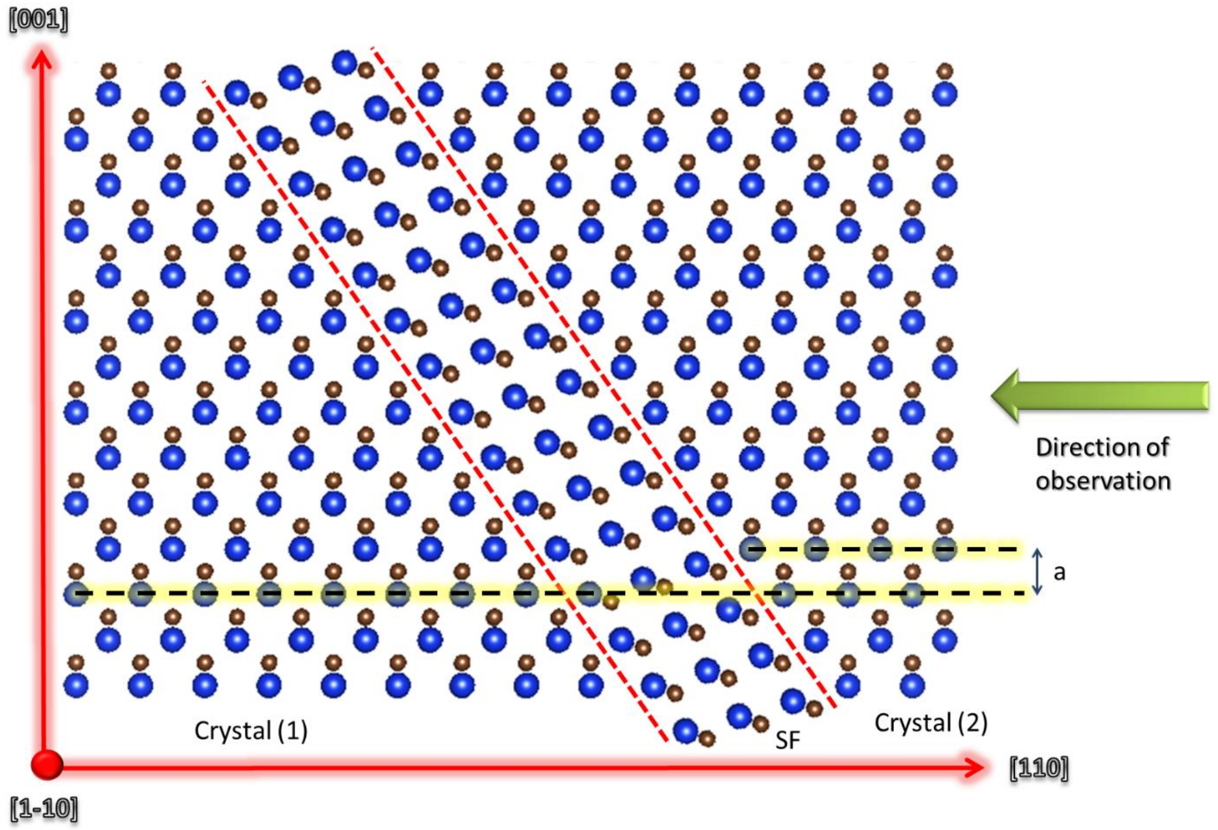

Figure S2. Atomic reconstruction was achieved with VESTA, which allowed to simulate two 3C-lattice domains delimited by a 6H-like SF. The Si atoms are represented by the blue spheres, while the C atoms are represented by the red spheres.

As highlighted by the black dashed lines, along this axis it can be seen how the crystal (1) on the left and the crystal (2) on the right of the defect are shifted by an entire lattice plane along the axis [001]. This shift preserves the alignment of the two crystals along the observation direction [110]. As a result, the HRSTEM image of the SF acquired along the zone axis [110] will show the usual stacking of the 3C-SiC lattice without introducing relative misalignments as displayed in the HRSTEM acquisition. This insight remarks how Figures 7d and 7f exhibit identical crystal arrangement despite the first was acquired on perfect 3C-SiC crystal and the second on 6H-like SF.
